# Permanent pacemaker implanted into patient’s left ventricle via subclavian artery by mistake: a case report

**DOI:** 10.1186/s12872-015-0032-2

**Published:** 2015-05-12

**Authors:** Guanmin Tang, Changlin Zhai, Zhiyong Wang, Hao Chen

**Affiliations:** Department of Cardiology, Jiaxing Cardiovascular Key Discipline, First Affiliated Hospital of Jiaxing University, No. 1882 South Zhonghuan Road, Jiaxing, Zhejiang 314000 China

**Keywords:** Permanent pacemaker, Left ventricle, Subclavian artery, Pacemaker electrode

## Abstract

**Background:**

Although various iatrogenic complications could be observed in the process of permanent pacemaker implantation, pacemaker electrode mistakenly implanted into left ventricle via subclavian artery and aortic valve has not been reported.

**Case presentation:**

Herein, we reported a 71-year-old woman with permanent pacemaker mistakenly implanted into the left ventricle. During the operation of permanent pacemaker implantation, puncture was performed on her subclavian artery by mistake, and then the pacemaker electrode was put into the cardiac apex of left ventricle via ascending aorta reversely.

**Conclusion:**

The further operation could be conducted.

## Background

Since the first successful implantation of permanent pacemaker in 1958, it has been widespread used in clinics and gradually becomes an important method to treat cardiovascular diseases [1]. Alternative pacing sites include traditional right ventricular apical and right ventricular septum, in which physiological pacing is stimulated. Up to now, both left ventricular epicardial pacing via coronary sinus and left ventricular endocardial pacing via the puncture site of atrial septum have been applied in clinics [2]. The iatrogenic complications of pacemaker implantation include pneumothorax, hemothorax resulting from entering the subclavian artery by mistake, electrode falling off, thromboembolism, cardiac perforation, cardiac tamponade etc. [3]. However, it has not been reported that the permanent pacemaker was implanted in left ventricular apex by mistake via subclavian artery. Herein, we reported the first case of such situation.

## Case presentation

A 71-year-old female patient who was diagnosed as “rheumatic valvular heart disease, mitral stenosis and insufficiency” underwent mitral valve replacement in our thoracic surgery department two years ago. After the operation, amiodarone (0.2 mg bid) was administrated to the patient for a long time because of paroxysmal atrial fibrillation. Four days before admission, she suffered from dizziness without syncope or amaurosis, and then came to our emergency room. Electrocardiogram (ECG) indicated atrial fibrillation with slow ventricular rates (37–38/min). Cardiac ultrasound demonstrated postoperative state of mitral valve replacement, enlargement of left atria and mild reflux of pulmonary valve. A temporary pacemaker was implanted before admission. The patient was diagnosed as “rheumatic valvular heart disease, postoperative state of mitral valve replacement, atrial fibrillation with slow heart rate, the state of temporary pacemaker implantation, chronic heart failure (NYHA IIclass), and diabetes”.

Amiodarone was discontinued after admission. One week later, we regulated the rhythm of patient’s temporary pacemaker to 30/min. ECG monitor suggested the occurrence of significant sinus bradycardia with atrio-ventricular junctional escape beat, and occasionally with pacing rhythm. Considering the degradation of patient’s sinuatrial node and atrioventricular node, a permanent dual-chamber pacemaker was prepared to be implanted. During the course of operation, patient suffered from chest distress, dyspnea, lung rales, and rapid atrial flutter in ECG, which suggested the aggravation of heart failure. After the treatment of oxygen, morphine (5 mg iv), and furosemide (20 mg iv), the patient was getting better. After operation, excess bleeding was found on the puncture site. Considering the severity of patient’s disease and poor operation endurance, a single-chamber pacemaker (PhilosIISR, BIOTRONIK Company) was chosen to take place of dual-chamber pacemaker after contacting with patient’s relatives. Parameters settings of the single-chamber pacemaker were demonstrated as follows: the threshold of ventricular pacing was 0.8 V (0.5mS); the amplitude of R wave was 30.1mv; and the electrode impedance was 856Ω.

**Fig. 1:**
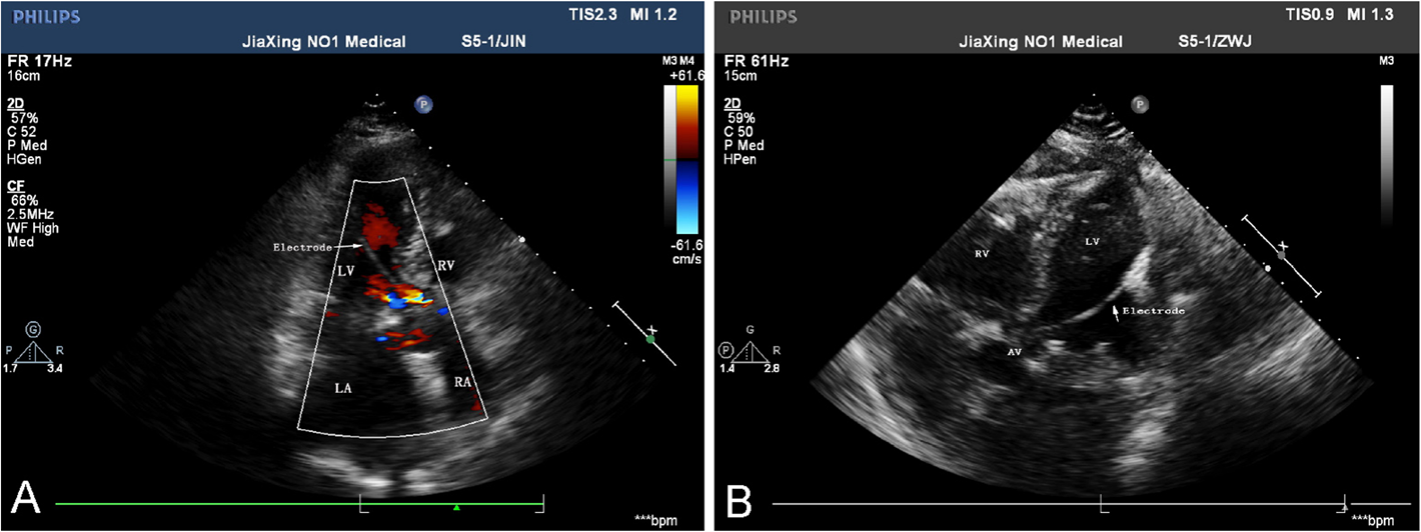
Cardiac ultrasound after operation. **A** Cardiac ultrasound demonstrated pacing electrode in ascending aorta and left ventricle. **B** Cardiac ultrasound demonstrated pacing electrode in ascending aorta and left ventricle

**Fig. 2:**
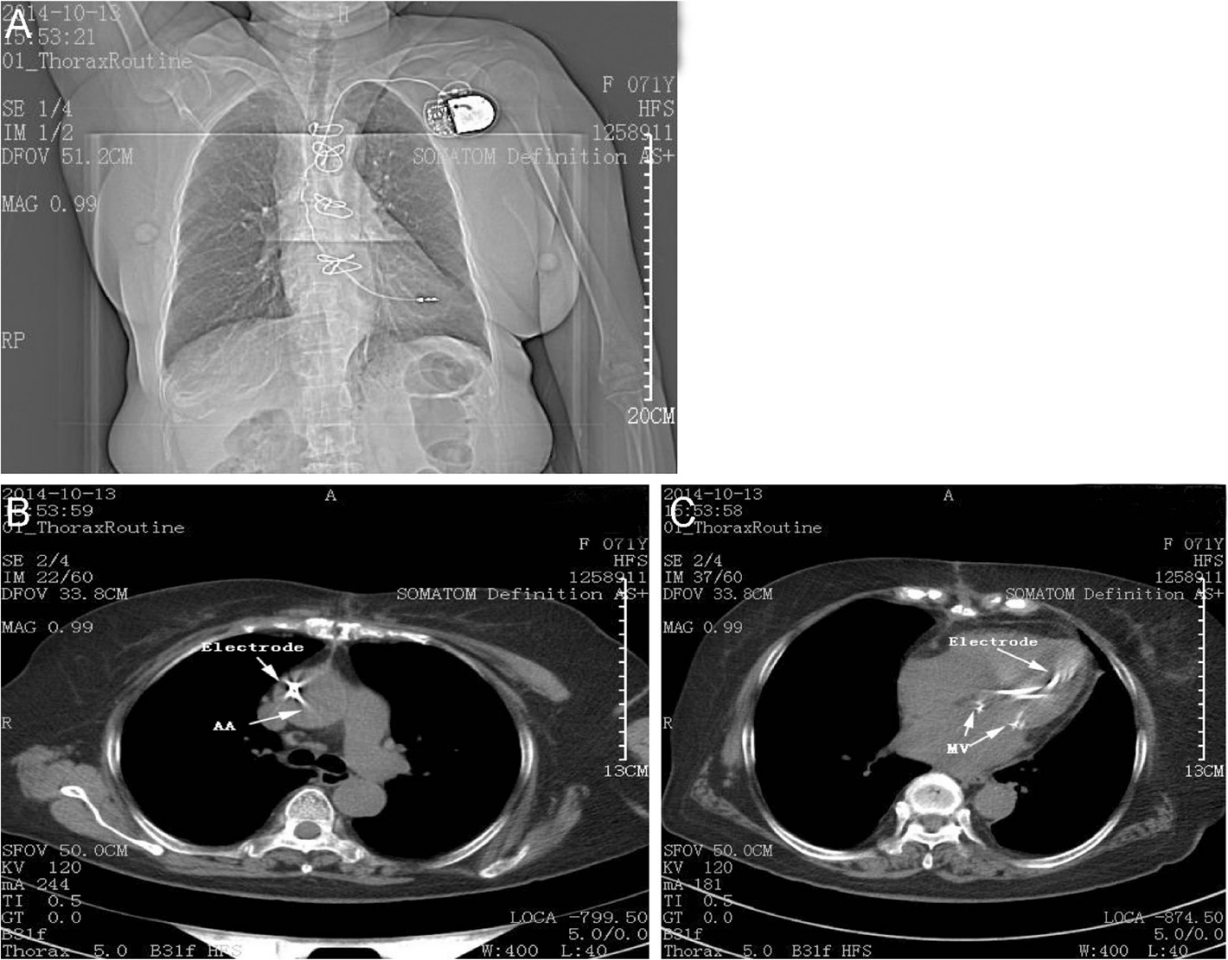
Chest CT after operation. **A** postoperative state of cardiac pacemaker implantation. **B** Chest CT scanning indicated pacing electrode in ascending aorta. **C** Chest CT scanning indicated the pacing leads in left ventricle

Both pacemaker function and Holter indicated favorable behavior of the pacemaker after the operation, and stitches were taken out one week later. Nine days later, the reexamination of cardiac ultrasound demonstrated as follows: postoperative state of mitral valve replacement, normal artificial biological mitral valve, enlargement of left atria, mild reflux of aortic valve, mitral valve flow spectrum showing a single peak, and pacemaker in ascending aorta and left ventricle (Fig. 1a, b). Chest computed tomography (CT) scan indicated that the pacemaker electrode was put into aorta and left ventricular (Fig. 2b, c), which suggested that the pacemaker had been put into subclavian artery by mistake and the pacing electrode was implanted in the left heart.

Traditional right ventricular apical pacing belongs to unphysiological pacing, while the most studied physiological pacing regions include right ventricular outflow tract (RVOT) and septum, which can improve patients’ heart function [4, 5]. However, various complications may appear during the course of RVOT and right ventricular septum pacing and increase the risk of operation, such as high threshold of pacing, difficult fixation and location of pacing leads, long-duration operation, easy dislocation, and even the cardiac perforation etc. [6–8]. Cardiac resynchronization therapy (CRT) has been widely applied in clinics to conduct left ventricular epicardial pacing via coronary sinus currently. However, it is difficult to conduct left ventricular endocardial pacing via the puncture site of atrial septum because of the occurrence of side-effects, such as high risk of thromboembolism, impact on the function of mitral valve, and myocardial damage caused by taking out the pacemaker. Therefore, we reported a case that pacemaker was implanted into left ventricle by mistake which made unnecessary troubles to our doctors.

**Fig. 3:**
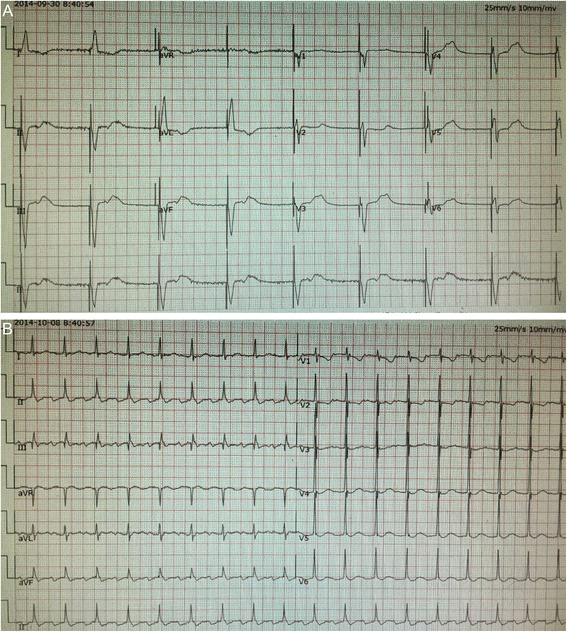
ECG after pacemaker implantation. **A** The left bundle-branch block in ECG after temporary pacemaker implantation. **B** ECG after permanent pacemaker implantation suggested rapid atrial flutter

In this case, patient’s diagnosis was definite. On October 6, 2014, a cardiac permanent pacemaker was implanted because the patient was suffering from atrial fibrillation and atrial flutter with sinus bradycardia (30-40/min). The reexamination of cardiac ultrasound demonstrated that the pacing leads were put into left heart, which suggested that pacing electrode was implanted into left ventricle via subclavian artery by mistake. The reasons included: 1) during the course of operation, patient’s heart function aggravated suddenly. Excess bleeding was observed after we performed a puncture on her subclavian artery by mistake. However, we regarded the excess bleeding and the increase of blood pressure as the results of the rise of central venous pressure caused by the aggravation of heart failure. Moreover, acute heart failure could result in hypoxia, which made troubles in recognizing whether the blood was from artery or vein; 2) after the pacing leads were implanted, the fluoroscopy should have been conducted in post-anterior view, 45-degree left anterior oblique view and 30-degree right anterior oblique view, respectively [9]. However, after the occurrence of chest distress, drugs were administrated to treat acute heart failure immediately, which also induce the operator to shorten the duration of operation. In addition, angiography has not been performed to confirm the location of pacing leads, and multiple body position fluoroscopy wasn’t conducted as well after the implantation of pacing electrode, while post-anterior view scanning during the course of operation couldn’t confirm the location of pacing electrode accurately (Fig. 2a); 3) it was easy to locate the passive electrode in traditional right ventricular apical pacing. In addition, the temporary pacing is implanted in the right ventricular apex generally, which is relatively helpful to locate the passive electrode in the right ventricular apex also. However, in our operation, the active electrode was used and prepared to implant into ventricular septum, which was difficult to locate. And it was hard to be located with the reference of the temporary pacemaker, which was normally positioned in the right ventricular apex. Therefore, we unfortunately mistook left ventricle for right ventricular low septum, and then the pacing electrode was implanted into left ventricle; 4) the electrocardiogram (ECG) after the pacemaker implantation is helpful in locating pacemaker. For example, the left bundle-branch block showed in ECG is common seen in right ventricular paced patients (Fig. 3a), while the right bundle-branch block in left ventricular paced patients. In this case, the patient suffered a sudden heart failure. The ECG suggested rapid atrial flutter without any pacemaker signal (Fig. 3b). Therefore, it was unable to evaluate the position of the lead through ECG.

## Conclusions

Further solutions included: 1) another operation would be performed to take out the pacing leads, and new cardiac permanent pacemaker should be implanted again. However, the risk of operation couldn’t be eliminated, and the biggest risk was the bleeding from the puncture site on subclavian artery. In order to prevent the occurrence of excess bleeding, covered stent or surgical ligation could be used, while the wound resulted from surgical ligation would be larger than that from covered stent; 2) Left ventricular permanent pacemaker could be preserved, and this might promote the occurrence of thromboembolism. Therefore, patients required life-long anticoagulant drugs. At the same time, pacing leads preserved in left heart might influence the function of aorta valve, lead to aortic insufficiency induced heart failure, or even result in the occurrence of cardiac perforation. The further operation could be conducted in our hospital or superior hospitals.

At last, there are several methods to early recognize and avoid the occurrence of such incidents: 1) When subclavian venous puncture is attempted, the subclavian artery is probably often advertently punctured. However, it can be distinguished by observing the color and pressure of the extraction blood. When necessary, arteriography can be adopted; 2) The successful venipuncture can be confirmed by observing the guidewire advancing into the inferior vena cava under fluoroscopy; 3) If ventricular premature beats with right bundle branch block happened frequently after the guidewire advanced into the ventricle, then the possibility of the guidewire entering the left ventricular through the subclavian artery need to be concerned; 4) After the pacemaker was implanted, right ventricular pacing was indicated when ECG showed the left bundle branch block, otherwise left ventricular pacing was indicated when ECG showed the right bundle branch block. 5) For a well-skilled operator, the technology of program-controlled telemetry for pacemakers and multi-dimensional images may help identify position of the pacing lead.

## Consent

Written informed consent was obtained from the patient for publication of this Case report and any accompanying images. A copy of the written consent is available for review by the Editor of this journal.

## Abbreviations

ECG: Electrocardiogram
CT: Computed tomography
RVOT: Right ventricular outflow tract
CRT: Cardiac resynchronization therapy
